# Hospital Festivals and Meetings

**Published:** 1895-02-16

**Authors:** 


### HOSPITAL FESTIVALS AND MEETINGS.

### ST. GEORGE'S HOSPITAL.

A special court of governors of St. George's Hospital was held on Friday, February 8th, (1) "To consider, and if approved, to empower the weekly board to expend a sum not exceeding £10,000 in providing a second operating theatre, in altering and rearranging the existing theatre, and in extending and otherwise altering the building under the operating theatre, viz., the chapel, the board-room, and the rooms in the basement." (2) "To consider, and if approved, to empower the weekly board to expend a sum not exceeding £18,000 in rebuilding and extending the nurses' home in Montpelier Street, Brompton." The first of these propositions met with objections from Mr. H. Maudslay, who said he considered the present an unfortunate time to embark on such an expenditure, when the hospitals of London were everywhere in a state of impecuniosity. Mr. Timothy Holmes, the treasurer, explained that the present housing of the nurses and servants left much to be desired, they having to dine in the basement, in a large hall common to them both. An adequate sick-room was very much needed. The alterations to the chapel, &c., and the new theatre were urgent. The money for these improvements would be partially provided for by a new legacy just announced. With regard to the nurses' home, the experiment of placing some of the nurses in three houses, which belonged to the hospital, was first tried some years ago, and had met with opposition on the score of its being undesirable for the nurses to have to go through the street on their way to and fro, but the plan had been found to work successfully. Now it was proposed to secure two more of these houses, the property of the hospital, pull them down, and build a proper nursing home. Mr. Agar Ellis condemned the expenditure as "wild and reckless." He urged the governors to consider before deciding upon this action. He strongly advocated waiting for the present until it might be possible to build upon their own site. He could not see there was any hardship in the nurses having to dine in the basement, nor did he see that a separate kitchen for the nurses was at all necessary. The Earl of Cork and Orrery supported the resolution, and expressed the opinion that if the fact of the money being needed for such a purpose were to be made widely known in the very rich neighbourhood surrounding the hospital, they would obtain plenty of outside help. Eventually both the resolutions were carried almost unanimously.

### ST. JOHN'S HOSPITAL FOR DISEASES OF THE SKIN.

The annual meeting of the governors of St. John's Hospital took place on February 6th, at the Hotel Victoria, Northumberland Avenue. The Earl of Chesterfield, president of the hospital, was in the chair. The report showed that the receipts for the year just closed were slightly in excess of those of the previous year—£3,645 1s. 1d., as against £3,597 3s. 3d., whilst there had been a decrease in the expenditure of £101. The debts owing by the hospital have also been reduced, now amounting to £510 2s. 10d. A special fund has been opened to defray the cost of certain necessary structural improvements, for which the sum of £1,500 will be required; £141 towards this is already in hand. The alterations in question are seriously needed, the accommodation both for the doctors and for the matron being very inadequate at the present time. During the year certain changes have been carried out in the nursing department, and a fully-trained matron and two charge nurses have been appointed. The number of both in-patients and out-patients attended has increased. The advantages now offered by St. John's Hospital as a school of dermatology appear to have been appreciated and the attendances at the various lectures have been good. Mr. Bertram Van Praagh testified to the urgent need for the contemplated improvements. He urged those who were interested in the hospital to visit it and see for themselves the work it was doing, and to help by contributions, so that one item of expense, that of advertising for funds, might be at least reduced. In moving a vote of thanks to the honorary medical staff, Mr. Josiah Oldfield spoke in warm terms of the ungrudging labour of the doctors, including a particularly cordial recognition of the work of the nursing staff.

### CITY OF LONDON TRUSS SOCIETY.

This society held its annual general meeting at 35, Finsbury Square, on Wednesday, February 6th, Mr. John Norbury, treasurer, in the chair. The report showed the general income for the past year to be £50 in excess of that for 1893, though this amount included but one legacy as against six in 1893. During the year the surgical staff had been increased in consequence of a larger number of patients, Mr. W. McAdam Eccles, F.R.C.S., being appointed as assistant surgeon. The patients treated in 1894 numbered 10,033. Many had been the benefits conferred by the supply of well-constructed instruments, and by the skilled treatment of the society's surgeons. Improvements had been effected as regards the patients' waiting-rooms and entrances, which were much appreciated. The report having been adopted, Sir Thomas Fowler, Bart., was elected trustee in the place of Mr. Alfred Lyon. At a special meeting which followed, a resolution was passed to sell out £2,000 funded stock, in order to discharge certain loans, and to meet increasing expenditure.

